# Gender differences in specialty preference among medical Students at Aleppo University: a cross-sectional study

**DOI:** 10.1186/s12909-020-02081-w

**Published:** 2020-06-05

**Authors:** Malke Asaad, Obada Zayegh, Joud Badawi, Zina shikh Hmidi, Ahmad Alhamid, Mario Tarzi, Sarab Agha

**Affiliations:** 1 Aleppo University, Faculty of Medicine, Aleppo, Syria; 2 Department of Pathology, Aleppo University, Faculty of Medicine, Al-Mouhafaza Aleppo, Syria

**Keywords:** Gender, Gender differences, Specialty, Medical Students, Residency preferences, Medical education

## Abstract

**Background:**

This study aims to identify gender differences in specialty preference and career choice among Syrian medical students.

**Method:**

A cross-sectional study comprising currently enrolled second, fourth and sixth year medical students at Aleppo University was conducted. Demographics, specialty preferences and factors influencing this decision were collected and analyzed.

**Results:**

A total of 561 students (44% males, 56% females) responded to our survey (87% response rate). Surgical specialties (40%) and internal medicine (16%) comprised the two most common specialties chosen by males. In contrast, the majority of females preferred other specialties (17%), internal medicine (16%) and surgical specialties (15%). The most common factor affecting the choice of a specialty by both genders (74% females and 71% males) was ‘A specialty that I like and find interesting’. Work/life balance and anticipated income were the second most common influencing factors by females (62%), males (67%), respectively. The majority of both genders supported the idea that medical students should be able to pursue any medical specialty they want, regardless of their gender. However, females more often believed that they had decreased opportunities for professional advancement based on their gender compared to males (33% vs. 4% respectively, *p* < 0.001).

**Conclusions:**

We illustrated significant gender differences in specialty preferences and factors influencing this decision. While the majority of participants agreed that medical students should be able to pursue any medical specialty they desire regardless of gender, more women believed they had decreased opportunities for professional advancement based on gender. Policy makers should advocate for a culture of gender equity and develop educational programs to insure gender balance of physicians into different specialties.

## Background

The percentage of female medical students has demonstrated a significant increase over the last few decades. Women constituted 46% of medical graduates in 2016 compared to 7% in 1966 according to the Association of American Medical Colleges (AAMC) [1]. Although the number of female medical students is comparable to their male counterparts, the scenario changes when analyzing the distribution of residents across various medical specialties. Only 15% of *orthopaedic surgery* and 17% of *neurological surgery* residency spots in the United States were occupied by females [2]. On the other hand, males constituted only 17% of *obstetrics and gynecology* and 29% of *pediatrics* residents [2]. These numbers reflect inherent gender segregation in specialty preference among medical students [3].

The medical educational system in Syria comprises 6 years of undergraduate medical education that commences following high school. The first year is a preparatory year which is a bridge to either medicine, dentistry, or pharmacy. The second and third years cover the basic sciences while the fourth and fifth year cover the clinical subjects. The final year is dedicated to clinical rotations, at the end of which, passing a national standardized exam is required to obtain the medical diploma. Residency spots are allocated according to applicants’ preferences and their scores obtained during medical school and that on the standardized exam.

Choosing a future specialty is a critical decision that medical students have to make at the end of medical school. It represents a major transformation in the education of these students from a broad exposure to a wide range of medical specialties to a specialized area of medical practice [4]. Several studies have assessed personality types and factors associated with medical students’ choice of subspecialty training [5–7]. In a systematic review of 75 studies from different populations, Yang et al. identified academic interests, competencies, and controllable lifestyles/ flexible work schedules to be the three most important factors driving the choice of a particular specialty among medical students [7]. Gender seems to play a significant role in the choice of a medical specialty and the factors associated with this decision.

The choices made by medical graduates regarding their future career are of prime importance due to their role in shaping the outlook of the healthcare system and ensuring an adequate medical workforce in a particular specialty [3, 5, 8]. Sawaf et al. demonstrated that males are more likely to study and work abroad compared to females [8]. This emphasizes the role of female doctors in balancing the gap created by the immigration of male doctors. Understanding the gender differences and factors influencing medical students’ specialty preference can help policy makers develop education programs and recruitment plans which can ensure balanced flux of physicians into different specialties [3, 5]. Although several studies have assessed factors influencing students’ choice of a particular specialty in different countries, no studies have evaluated the role of gender in specialty choice among Syrian medical students. The goal of this study is to identify gender differences in specialty preference, factors influencing the choice of subspecialty training and gender equity in career choice among medical students at Aleppo University.

## Methods

### Participants and data collection

A cross-sectional survey study was conducted to assess factors associated with specialty preference and gender equity. The study cohort comprised currently enrolled second, fourth and sixth year medical students at Aleppo University. We excluded students from the first year since this is a preparatory year which acts is a bridge to either medicine, dentistry, or pharmacy. Given that students in their 2nd and 3rd; 4th and 5th years have similar subjects (basic and clinical science, respectively), the inclusion of 2nd, 4th, and 6th years constitutes a representative sample across all years. Three random days were selected in April 2019, during which the survey was administered by the study team. Students in their 2nd, 4th, and 6th years, were approached during the 3 days of survey administration following class lectures, after clinical rotations, in the medical library, and on an individual basis. This took place at two sites: the Faculty of Medicine and Aleppo University Hospital. The total number of medical students in the included years was 2425. A power analysis was not performed.

Students were informed of the study goal and that participation is voluntary and anonymous. All questions pertaining to the study were answered and verbal consent was obtained. Students were handed a paper copy of the survey for completion.

### The survey

The questionnaire was built based on review of pertinent literature [3, 5, 9–11]. Expert feedback and discussion groups provided feedback regarding the survey style, content, and the length. The survey was piloted on 10 medical students (6 males, 4 females) and was found to be of appropriate length, clarity and content. Therefore, no subsequent changes were made. The survey was divided in three sections. The first section assessed students’ demographics, including age, gender, average score in medical school up to the time of survey administration, preferred employment status, and parents’ education level and employment status. The second section inquired about preferred specialty, and factors influencing career preference. Students were asked to choose one out of five categories for the preferred specialty, which included the following: surgical specialties (general surgery, orthopedics, plastic surgery, neurosurgery, urology, cardiothoracic surgery); internal medicine (general internal medicine, cardiology, GI, pulmonology, nephrology, neurology, rheumatology, endocrinology, hematology/oncology, infectious disease); pediatrics; OB/GYN; and other (dermatology, ENT, ophthalmology, radiology, anesthesiology, pathology, laboratory medicine, emergency medicine, psychiatry, family medicine, other). Students who had not decided which career path they will be pursuing were given the option to choose so. Participants were asked to mark all factors associated with their decision to pursue specific specialty, and to rank the top three factors associated with their decision.

The last section addressed gender equity in specialty preference. Students were asked whether medical students should pursue any specialty they want regardless of their gender, whether they suffered preferential treatment, and if they have different opportunities for professional advancement based on gender. We also inquired about priorities and gender roles in a relationship. The survey was first developed in English then translated to Arabic by (MA and OZ). The survey was administered in Arabic which is the native language and the language of medical education in Syria. An English version of the survey is presented in Additional file 1. This study was approved by the dean of the Faculty of Medicine at Aleppo University and complied with the Declaration of Helsinki.

### Statistical analysis

Categorical variables were presented as percentages and proportions while continuous data was summarized as mean and standard deviation (SD). To identify differences between males and females, Chi-square test was used to compare categorical variables, while continuous variables were analyzed using t-test. A *p*-value of < 0.05 was considered statistically significant. All statistical analysis was performed using JMP Pro 14 software (JMP, Pro 14, SAS Institute Inc., Cary, NC, 1989–2019).

## Results

Out of 644 medical students, a total of 561 (44% males, 56% females) responded to our survey (87% response rate). Gender data was absent for 2 students. The mean age was 21 years (SD, 2) and was similar between the two genders. The majority of respondents were second year medical students (42%), followed by fourth (30%) and sixth (28%). No significant differences were identified between males and females in regards to average score in medical school, marital status, and father’s educational level. However, mothers of female students had significantly higher education levels compared to males’ students (65% of females’ mothers had a college or university degree compared to 50% in males). Female students preferred less working hours (68% preferred ≤6 working hours per day and 28% ≥7 h per day) compared to males (45% chose ≥7 and ≤ 6 equally). Table 1 demonstrates student demographics.

**Table 1:** Student demographics

	Male N(%)	Female N(%)	Total	***p-value***
**Number of respondents**	245	314	561	
**Age, years**	21 (2)^b^	21 (2)^b^	21 (2)^b^	0.542
**Year of medical school**				
Second	113 (46)	122 (39)	235 (42)	0.228
Fourth	67 (27)	100 (32)	169 (30)	
Sixth	65 (27)	91 (29)	156 (28)	
**Average score in medical school**				
< 80	120 (50)	147 (48)	269 (49)	0.605
≥ 80	121 (50)	162 (52)	283 (51)	
**Marital status**				
Single	228 (93)	293 (93)	522 (93)	0.932
Married	3 (1)	5 (2)	8 (1)	
Other	13 (5)	16 (5)	30 (5)	
**Father’s level of education**				
Less than high school	48 (20)	46 (15)	96 (17)	0.406
High school	28 (12)	44 (14)	72 (13)	
College	24 (10)	39 (13)	63 (11)	
University	118 (49)	159 (51)	277 (50)	
Physician	22 (9)	24 (8)	46 (8)	
**Mother’s level of education**				
Less than high school	80 (33)	66 (21)	148 (27)	0.006^a^
High school	39 (16)	42 (13)	81 (15)	
College	33 (14)	63 (20)	96 (17)	
University	75 (31)	125 (40)	200 (36)	
Physician	13 (5)	16 (5)	29 (5)	
**Father’s work hours per day**				
No work	32 (14)	33 (11)	65 (12)	0.544
≤ 6	61 (26)	77 (25)	139 (25)	
≥ 7	144 (61)	199 (64)	344 (63)	
**Father’s work, days per week**	5 (2)^b^	5 (2)^b^	5 (2)^b^	0.129
**Mother’s work, hours per day**				
No work	137 (58)	165 (54)	303 (55)	0.161
≤ 6	59 (25)	98 (32)	157 (29)	
≥ 7	42 (18)	44 (14)	87 (16)	
**Mother’s work, days per week**	2 (3)^b^	2 (3)^b^	2 (3)^b^	0.688
**Preferred employment status, hours per day**				
No work	23 (9)	12 (4)	35 (6)	< 0.0001^a^
≤ 6	110 (45)	209 (68)	321 (58)	
≥ 7	110 (45)	88 (28)	198 (36)	
**Preferred employment status days per week**	5 (2)^b^	5 (1)^b^	5 (1)^b^	0.55

^a^Statistically significant; ^b^Mean (standard deviation)

### Specialty preference

**Fig. 1:**
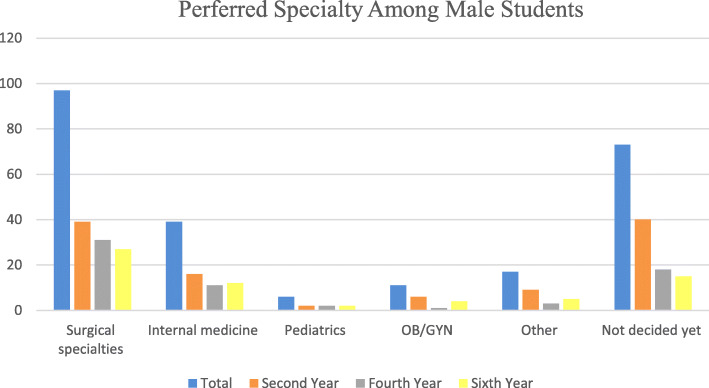
Preferred Specialty Among Male Students

**Fig. 2:**
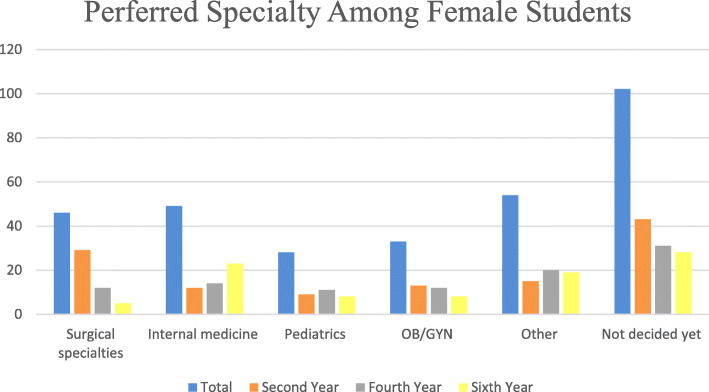
Preferred Specialty Among Female Students

Significant differences were identified between males and females regarding the specialty of choice. Surgical specialties (40%) and internal medicine (16%) comprised the two most common specialties chosen by males. In contrast, the majority of females preferred other specialties (17%), internal medicine (16%) and surgical specialties (15%). 33% of females and 30% of males were not decided regarding their career specialty. As demonstrated in Figs. 1 and 2, the percentage of male and female students pursuing surgical specialties decreased for students in the latter years of their medical education. Similar pattern was found for undecided students. These trends were significant for female students (*p* = 0.005), unlike their male counterparts (*p* = 0.675).

The three most common factors affecting specialty choice according to females were ‘A specialty that I like and find interesting’ (74%), ‘Balance between work and being a good parent/raising child’ (62%), ‘Personal values and ambition’ (53%). For males, ‘A specialty that I like and find interesting’ (71%), ‘Anticipated income’ (67%), ‘Personal values and ambition’ (47%) comprised the three most common influencing factors. When asked to rank the three most important factors that could play a role in their specialty choice, ‘A specialty that I like and find interesting’ was the most common factor to be ranked 1 by both males (19%) and females (21%). ‘Balance between work and being a good parent/raising child ranked’ was the most common factor to be ranked second for females (13%), while ‘Anticipated income’ was the most common factor to be ranked second (16%) by males. Table 2 summarizes preferences and influencing factors of the surveyed students. More males reported that the conflict in Syria affected their choice of specialty (31%), compared to female students (20%).

**Table 2:** Specialty preference

	Male N(%)	Female N(%)	Total	***p-value***
**Preferred Specialty**				
Surgical specialties	97 (40)	46 (15)	144 (26)	< 0.0001^a^
Internal medicine	39 (16)	49 (16)	88 (16)	
Pediatrics	6 (2)	28 (9)	34 (6)	
OB/GYN	11 (5)	33 (11)	44 (8)	
Other	17 (7)	54 (17)	71 (13)	
Not decided yet	73 (30)	103 (33)	177 (32)	
**Factors**				
On-call schedule	49 (20)	104 (33)	153 (27)	0.0006^a^
Hours of work	77 (31)	103 (33)	180 (32)	0.73
Duration of residency	84 (34)	96 (31)	182 (32)	0.351
Not requiring much physically exertion	50 (20)	129 (41)	180 (32)	< 0.0001^a^
Balance between work and being a good parent/raising child	94 (38)	195 (62)	290 (52)	< 0.0001^a^
I want to give time to my friends, family (spouse, parents, other family members) and hobbies.	110 (45)	162 (52)	272 (48)	0.116
Anticipated income	165 (67)	135 (43)	301 (54)	< 0.0001^a^
Prestige of that specialty/ specialty with high social status	49 (20)	42 (13)	92 (16)	0.035^a^
A specialty that I like and find interesting	175 (71)	232 (74)	409 (73)	0.517
Career prospects, specialty that would achieve my life goal	111 (45)	122 (39)	233 (42)	0.125
Specialty in line with technical skills (that requires Talent for specific skill)	86 (35)	69 (22)	156 (28)	0.0006^a^
Intellectual content of the specialty (Intellectual challenge)	83 (34)	81 (26)	165 (29)	0.037^a^
Having a family member from that specialty	10 (4)	11 (4)	21 (4)	0.721
Advice from family members	20 (8)	47 (15)	67 (12)	0.014^a^
Advice from spouse/future spouse	18 (7)	41 (13)	60 (11)	0.029^a^
Advice from mentor, a teacher	22 (9)	32 (10)	54 (10)	0.667
Interaction with physicians from same gender	18 (7)	16 (5)	35 (6)	0.269
Interaction with residents from same gender	12 (5)	17 (5)	29 (5)	0.785
Type or gender of the patient in that specialty	19 (8)	34 (11)	53 (9)	0.219
Less exposure to patients	27 (11)	17 (5)	44 (8)	0.015^a^
I chose a specialty needed by the community (Community needs more experts in this specialty).	98 (40)	109 (35)	208 (37)	0.199
Personal life experience	59 (24)	70 (22)	129 (23)	0.619
The teachings of my religion/philosophy have a role in choosing this specialization.	26 (11)	50 (16)	76 (14)	0.069^a^
Society view of physician gender in this specialty.	22 (9)	54 (17)	76 (14)	0.005^a^
Personal values and ambition.	115 (47)	167 (53)	283 (50)	0.143

^a^Statistically significant

### Gender equity

The majority of both males (44%) and females (44%) agreed that women have superior advantage over men in some medical specialties; while men have superior advantage over women in some medical specialties. Similarly, the majority in both genders (56% of males, and 60% of females) strongly supported the idea that medical students should be able to pursue any medical specialty they want, regardless of their gender. Almost half of respondents belonging to either gender reported more comfort working with a mentor, colleague and patients from the same gender. Females were more likely to believe that they have decreased opportunities for professional advancement based on their gender compared to males (33% vs. 4% respectively, *p* < 0.001). Males expressed that they suffered from preferential treatment in their medical training based on gender more often than females (24% of males expressed suffering from preferential treatment every day/several times per month compared to 14% of females, *p* = 0.009).

More females (77%) believed in equal value of both partners’ career in a relationship compared to males (56%), *p* < 0.0001. Similarly, more females believed that both career and family are of equal priorities (68%) compared to males (51%), *p* < 0.0001 (Table 3).

**Table 3:** Gender equity in medical specialties

	Male N(%)	Female N(%)	Total	***p-value***
**In medicine:**				
Men have superior advantage (men treated better) over women in all medical specialties	12 (5)	12 (4)	24 (4)	0.234
Men have superior advantage (men treated better) over women in most medical specialties	43 (18)	60 (19)	103 (18)	
Women have superior advantage (women treated better) over men in all medical specialties	2 (1)	3 (1)	5 (1)	
Women have superior advantage (women treated better) over men in most medical specialties	10 (4)	3 (1)	13 (2)	
Women have superior advantage (women treated better) over men in some medical specialties; while men have superior advantage (men treated better) over women in some medical specialties. There is no advantage based on gender.	108 (44)	138 (44)	248 (44)	
There is no advantage based on gender.	68 (28)	97 (31)	165 (30)	
**Medical students should be able to pursue any medical specialty they want, regardless of their gender.**				
Strongly support	137 (56)	186 (60)	324 (58)	0.193
Somewhat support	64 (26)	83 (27)	148 (27)	
Somewhat oppose	35 (14)	40 (13)	75 (13)	
Strongly oppose	7 (3)	2 (1)	9 (2)	
**In your medical training, how often do you experience preferential treatment based on gender**				
Every day/ several times per month	56 (24)	44 (14)	100 (18)	0.009^a^
Once a month/ several times per year	60 (25)	73 (24)	134 (25)	
Once a year/ not at all	120 (51)	190 (62)	311 (57)	
**In your medical training, how often do you observe preferential treatment based on gender**				
Every day/ several times per month	113 (47)	91 (30)	204 (37)	< 0.001^a^
Once a month/ several times per year	65 (27)	98 (32)	164 (30)	
Once a year/ not at all	60 (25)	117 (38)	178 (33)	
**Do you feel more comfortable working with a mentor of the same gender? (yes)**	133 (55)	154 (50)	288 (52)	0.277
**Do you feel more comfortable working with a colleague of the same gender? (yes)**	161 (67)	197 (64)	359 (65)	0.417
**Do you feel more comfortable working with a patient of the same gender? (yes)**	120 (51)	151 (50)	272 (50)	0.862
**The conflict in this country has affected my choice of specialty. (yes)**	75 (31)	61 (20)	137 (25)	0.005^a^
**In my professional career:**				
I have increased opportunities for professional advancement based on my gender	64 (28)	14 (5)	78 (15)	< 0.001^a^
I have decreased opportunities for professional advancement based on my gender	9 (4)	97 (33)	106 (20)	
Gender plays no role in my professional advancement.	156 (68)	180 (62)	338 (65)	
**If you are currently married or plan to get married in the future, whose career takes/will take priority in the relationship?**				
My career	37 (15)	11 (4)	48 (9)	< 0.0001^a^
My partner’s career	16 (7)	24 (8)	40 (7)	
Both equally	135 (56)	238 (77)	374 (68)	
NA	51 (21)	37 (12)	89 (16)	
**Which of the following takes/will take priority in your life?**				
Your career	34 (14)	17 (6)	51 (9)	< 0.0001^a^
Your family and children	50 (21)	64 (21)	114 (21)	
Both equally	123 (51)	210 (68)	334 (60)	
NA	34 (14)	18 (6)	53 (10)	

^a^Statistically significant

## Discussion

Our study has revealed significant differences in specialty preferences and factors influencing career choice among medical students at Aleppo University. The majority of participants agreed that medical students should be able to pursue any medical specialty they desire regardless of gender. More women believed that they have decreased opportunities for professional advancement based on gender compared to men.

Gender differences in choosing a career specialty is a topic of current interest. Several studies have assessed these distinctions in different populations and showed that female students have a greater interest in pediatrics and obstetrics and gynecology compared to their male colleagues while surgical specialties were less preferred by female students [5, 8, 10, 12–14]. The percentage of female residents in different specialties in the United States as reported by the AAMC supports the findings of these reports [2]. This goes in line with our findings, where more males opted for surgical specialties and more females were interested in pursuing a career in pediatrics, obstetrics and gynecology and other specialties. Although we did not follow-up students during their medical school years, we noticed that the percentage of students seeking a career in surgery decreased as students advanced through their medical education. However, this decrease was more significant for females compared to males as shown in Figs. 1 and 2. This is similar to the findings by Khader et al. where 64% of 2nd year male students and 25% of 2nd year female students were interested in surgery compared to 34% of male students and 8% of female students in their sixth year [5]. This change may be the result of exposure to surgical specialties during clinical rotations. In Syrian medical schools, students are involved in surgical rotations in the fourth, fifth, and sixth years. Many students are influenced by the perceived social prestige of surgeons early in their medical education. However, as they get exposed to the challenges and lifestyle of surgical specialties, they may realize that other specialties would be a better fit for their future career.

Several factors could play a role in choosing a specialty of interest. In our study, ‘A specialty that I like and find interesting’ was the most common factor to affect the choice of a specialty and the most common factor to be ranked ‘1’ by both genders. When looking at the second most commonly chosen factor, gender differences were apparent. Females were more likely to choose work-life balance while males were more influenced by anticipated compensation. This is consistent with prior studies showing that women tend to choose specialties which will ensure adequate time for their families and children and males opt for specialties that will guarantee adequate income and social prestige [3, 5, 14, 15]. The fact that 68% of our female participants chose equal importance for family and career in their lives (compared to 51% of males) and the differences in preferred employment status found in our study (where females preferred less working hours compared to males) explain the differences in choosing certain influencing factors. However, these differences might be as result of gender segregation in specialties which are dominated by males or females. In our study, 44% of both genders agreed that women have superior advantage over men in some specialties; while men have superior advantage over women in other specialties. Gender bias exists for both genders, and discrimination has been reported by those belonging to the gender minority [9, 16, 17]. On the other hand, 33% of our female participants believed they had decreased opportunities for professional advancement based on gender compared to 4% of males; possibly reflecting gender bias against the professional advancement of females. Interestingly, females were less likely to report suffering from unequal treatment compared to males. This can be a result of benevolent sexism which encompasses subjectively positive attitude towards women endorsing stereotypical gender defined roles [9, 18].

Studies have shown that role models and personality types seems to play a critical role in choosing a specialty [6, 9, 10, 19]. Gargiulo et al. found that perceived surgical personality and surgical culture, not lifestyle and workload issues were the main deterrents to choosing a career in surgery for women [20]. In contrast, Jagsi et al. did not identify significant associations between exposure to role models and choice of specialty [21]. However, they observed that female students were more likely than males to enter residency programs with higher proportions of female residents [21]. In our study, ‘Interaction with physicians from same gender’ and ‘Interaction with residents from same gender’ was a minor contributing factor to specialty choice (5–7%).

Spreading awareness in the society and changing the current medical residency system are critical steps to improving gender equality in medicine. Gender roles and stereotypes which emphasize male responsibilities in working and female responsibility in taking care of the family and children should also be targeted in awareness programs advocating for gender equity due to its portrayal of an influence over the choice of medical specialties. Since interest was the top-ranked contributing factor of specialty choice for both males and females, stimulating students’ interests in certain specialties early in their medical education may help close the gender gap and improve the unequal distribution of physicians in certain specialties [7].

Our study is the first to assess gender differences in specialty choice and influencing factors among Syrian medical students. However, it has its several limitations. The survey used in this study is not a validated tool and was based on literature review and expert opinions which introduce inherent biases. Our results were derived from a sample from single medical school which may not be representative of the entire medical school and other Syrian universities. To identify transgenders and non-binaries, we added the choice ‘other’ to the gender questions. However, this was not chosen by any of the students which may be a results of the conservative nature of the Syrian society. The majority of students did not report the details of their choice of the exact specialty within the category or chose more than one subspecialty which precluded the possibility of further analyzing these results. Finally, we did not follow-up students to identify changes in their specialty choice or assess gender equity among current residents at our hospital. Future studies could assess gender differences among residents and how medical students’ opinions change as they advance in their medical education through a prospective design.

## Conclusion

We illustrated significant gender differences in specialty preferences and factors influencing this decision. While the majority of participants agreed that medical students should be able to pursue any medical specialty they desire regardless of gender, women more often believed that they had decreased opportunities for professional advancement based on gender compared to men. Moreover, most students believe that gender segregation exist in certain specialties. Policy makers should advocate a culture of gender equity and develop educational programs and recruitment plans to insure gender balance of physicians into different specialties and to improve the current healthcare system.

## Supplementary information

**Additional file 1.** Appendix 1.

## Abbreviation

AAMC: American association of medical colleges
