# Supplementary material for: Gender differences in specialty preference among medical Students at Aleppo University: a cross-sectional study
Source: BMC Med Educ. 2020 Jun 5;20:184. doi: 10.1186/s12909-020-02081-w (PMC7275529; doi:10.1186/s12909-020-02081-w)
Supplement: Supplementary file 1 — Additional file 1. Appendix 1. [file 12909_2020_2081_MOESM1_ESM.docx]

**Gender Equity and career choice among medical students at Aleppo University**

1. What is your age? ___________
2. What is your gender? 🞎 Male 🞎 Female 🞎 Other
3. Which year of medical school are you currently in?
   1. 🞎 First b. 🞎 Second c. 🞎 Third d. 🞎 Fourth e. 🞎 Fifth f. 🞎 Sixth
4. What is your marital status?
   1. 🞎 Single b. 🞎 Married c. 🞎 Other
5. What is your father’s level of education?
6. 🞎 Less than high school. b. 🞎 high school. c. 🞎 college. d. 🞎 university. e. 🞎 physician.
7. What is your mother’s level of education?
   1. 🞎 Less than high school b. 🞎 high school. c. 🞎 college. d. 🞎 university. e. 🞎 physician.
8. How many hours does your father work a day, and how many days per week?
   1. 🞎 <4 b. 🞎 4-6 c. 🞎 7-8 d. 🞎 >8 e. 🞎 no work.

Days per week: ___________

1. How many hours does your mother work a day and how many days per week?

a. 🞎 <4 b. 🞎 4-6 c. 🞎 7-8 d. 🞎 >8 e. 🞎 no work.

Days per week: ___________

1. What is your average score in medical school?
   1. 🞎 <60 b. 🞎 60-69 c. 🞎 70-79 d. 🞎80-89 e. 🞎 90-100.
2. What is your preferred employment status (hrs/day and days/week)?

a. 🞎 <4 b. 🞎 4-6 c. 🞎 7-8 d. 🞎 >8 e. 🞎 I prefer not to work.

Days per week: ___________

**Medical Specialty**

1. What is your preferred specialty? *Choose one option only for category and one option only for specialty*.
   1. 🞎 Surgical specialties (general surgery, orthopedics, plastic surgery, neurosurgery, urology, cardiothoracic surgery)
   2. 🞎 Internal medicine (general internal medicine, cardiology, GI, pulmonology, nephrology, neurology, rheumatology, endocrinology, hematology/oncology, infectious disease)
   3. 🞎 Pediatrics
   4. 🞎 OB/GYN
   5. 🞎 Other (Dermatology, ENT, ophthalmology, radiology, anesthesiology, pathology, laboratory medicine, emergency medicine, psychiatry, family medicine, other).
   6. 🞎 I have not decided yet but I am tending towards ___________
2. Which of the following factors do play or could play a role in choosing specialty of interest (select all that apply)
3. 🞎 On-call schedule
4. 🞎 Hours of work
5. 🞎 Duration of residency
6. 🞎 Not requiring much physically exertion
7. 🞎 Balance between work and being a good parent/raising child
8. 🞎 I want to give time to my friends, family (spouse, parents, other family members) and hobbies.
9. 🞎 Anticipated income
10. 🞎 Prestige of that specialty/ specialty with high social status
11. 🞎 A specialty that I like and find interesting.
12. 🞎 Career prospects, specialty that would achieve my life goal
13. 🞎 Specialty in line with technical skills (that requires Talent for specific skill)
14. 🞎 Intellectual content of the specialty (Intellectual challenge)
15. 🞎 Having a family member from that specialty
16. 🞎 Advice from family members
17. 🞎 Advice from spouse/future spouse
18. 🞎 Advice from mentor, a teacher
19. 🞎 Interaction with physicians from same gender
20. 🞎 Interaction with residents from same gender
21. 🞎 Type or gender of the patient in that specialty
22. 🞎 Less exposure to patients
23. 🞎 I chose a specialty needed by the community (Community needs more experts in this specialty).
24. 🞎 Personal life experience
25. 🞎 The teachings of my religion/philosophy have a role in choosing this specialization.
26. 🞎 Society view of physician gender in this specialty.
27. 🞎 Personal values and ambition.
28. 🞎 Other ___________
29. Rank the three most important factors from the previous question that play/could play a role in choosing specialty of interest.

**Gender Equity:**

1. Choose from the following, the one statement that you agree with the most. In medicine:
2. 🞎 Men have superior advantage (men treated better) over women in all medical specialties
3. 🞎 Men have superior advantage (men treated better) over women in most medical specialties
4. 🞎 Women have superior advantage (women treated better) over men in all medical specialties
5. 🞎 Women have superior advantage (women treated better) over men in most medical specialties
6. 🞎 Women have superior advantage (women treated better) over men in some medical specialties; while men have superior advantage (men treated better) over women in some medical specialties
7. 🞎 There is no advantage based on gender.
8. To what extent do you support or oppose following statement: Medical students should be able to pursue any medical specialty they want, regardless of their gender.
   a.🞎 strongly support b.🞎 somewhat support c.🞎 somewhat oppose d.🞎 strongly oppose
9. In your medical training, how often do you suffer from preferential treatment based on gender
   1. 🞎 Every day b.🞎 several times per month c.🞎 once a month d.🞎 several times per year.

e.🞎 once a year f.🞎 not at all.

1. In your medical training, how often do you observe preferential treatment based on gender
   1. 🞎 Every day b.🞎 several times per month c.🞎 once a month d.🞎 several times per year.

e.🞎 once a year f.🞎 not at all.

1. Do you feel comfortable working with a mentor of the same gender?

a. 🞎 Yes. b. 🞎 No.

1. Do you feel comfortable working with a colleague of the same gender?

a. 🞎 Yes. b. 🞎 No.

1. Do you feel comfortable working with a patient of the same gender?

a. 🞎 Yes. b. 🞎 No.

1. The conflict in this country has affected my choice of specialty

a. 🞎 Yes. b. 🞎 No.

1. Choose from the following, the one option that you agree with the most. In my professional career:
   1. 🞎 I have increased opportunities for professional advancement based on my gender
   2. 🞎 I have decreased opportunities for professional advancement based on my gender
   3. 🞎 Gender plays no role in my professional advancement.

22-If you are currently married or plan to get married in the future, whose career takes/will take priority in the relationship?

a. 🞎 My career b. 🞎 My partner’s career c. 🞎 Both equally d. 🞎 Not Applicable

23- Which of the following takes/will take priority in your life?

a. 🞎 Your career b. 🞎 Your family and children c. 🞎 Both equally d. 🞎 Not Applicable

Optional question: What is your suggestion to improve gender equity in medicine?
____________________________________________________________________________________________________________________________________________________________________________________________________________________________________________________________________________________________________________________________________________
